# Critical success factors for Vietnamese laboratories striving to implement quality management systems

**DOI:** 10.4102/ajlm.v9i1.937

**Published:** 2020-12-18

**Authors:** Cathy Robinson, James Johnson, Katy Yao, Hien Bui

**Affiliations:** 1 International Consulting Services, Louisiana State University Alexandria, Alexandria, Louisiana, United States; 2 School of Health Sciences, Central Michigan University, Mount Pleasant, Michigan, United States; 3 Division of Global HIV and TB Program, Centers for Disease Control and Prevention, Atlanta, Georgia, United States; 4 Centers for Disease Control and Prevention, Hanoi, Vietnam

**Keywords:** critical success factors, medical laboratories implementing QMSs, laboratories earning ISO 15189 accreditation, quality management system helps laboratories improve quality, accuracy, reliability of patient results

## Abstract

Accurate laboratory reporting is crucial to patient diagnosis and treatment. This study identified critical success factors (CSF) for implementing a laboratory quality management system (QMS). This descriptive research used qualitative and quantitative methods to collect and analyze data from laboratory managers and staff employed in Vietnamese hospital laboratories implementing a QMS. The top five CSFs identified were: (1) staff QMS knowledge, (2) manager leadership, (3) staff commitment, (4) mentorship, and (5) hospital administration support. Identifying CSFs is critical to successful planning and implementation of QMS.

## Introduction

Medical laboratories provide critical services which are used by physicians to accurately diagnose, treat and monitor patient health. Grycotis, *The Infectious Advisor*, emphasises the value of laboratory test results to clinicians in making an accurate diagnosis and monitor treatment regimes.^1^ Equally important is the detrimental effect inaccurate results have including wrong diagnosis, wrong treatment, and patient death. Likewise, Nkengasong et al., emphasise that quality laboratory systems are needed to achieve the Millennial Development Goals for health and meet universal access for treatment of HIV/AIDS, tuberculosis and malaria.^2^

Similarly, the International Standardization Organization (ISO) 15189 accreditation is recognised as the gold standard for measuring laboratory quality globally.^3^ One pathway to attaining this accreditation is by implementing a quality management system (QMS). In addition to a QMS training programme, the World Health Organization developed a checklist entitled Stepwise Laboratory Quality Improvement Process Towards Accreditation to measure quality improvement within laboratories and provide continued recognition and motivation of staff to continue and sustain improvements.^4^ An international effort was fielded in developing countries to educate and train laboratory management and staff on improving the accuracy and reliability of laboratory results. QMS training is designed to teach participants how to identify deficiencies in their laboratories, design improvement projects to fill those gaps, and enact standard practices and processes into daily laboratory practices. Following the training, managers and staff return to their respective laboratories to begin improvement. Intermittent self-assessments serve to monitor the improvement process, show improvements and motivate to continue improvement. Several previous studies^5,6,7^ have evaluated the outcome of these training programmes on laboratory quality by comparing laboratory assessments before and after QMS implementation. While this is a good first step, quantitative statistics do not identify ‘why’ a particular standard was or was not met. Adding a qualitative component allows for the identification of ‘why’ QMS implementation assessment scores often showed wide variability.

In 1979, Rockart worked with executives in manufacturing organisations to develop a set of factors aimed to guide goal development which proved to significantly contribute to project success. Rockart referred to these factors as critical success factors (CSFs).^8^ The primary benefit CSFs offer any organisation is the ability to focus organisation efforts for project success. However, there is a noticeable lack of published literature identifying CSFs for success in implementing QMS projects in medical laboratories. This study aimed to identify CSFs for medical laboratories implementing a QMS project and ‘why’ a laboratory’s score may or may not improve over time.

## Methods

### Ethical considerations

This research received approval from the Institutional Review Board at Central Michigan University. This activity was reviewed per the Centers for Disease Control and Prevention (CDC) human research protection procedures and determined to be a non-research, public health programme activity. The Vietnam Administration of Medical Services agreed to this study and wrote letter no. 1506/KCB-QLCL to each hospital granting data collection permission to the researcher. Written informed consent was obtained from all participants. Results were reported in aggregate form only.

### Study participants

The Vietnam Administration for Medical Services assisted in selecting four laboratories (three city level and one district level, coded as H1–H4) based on their managers’ and staffs’ willingness to share their experiences while implementing a QMS project. Each laboratory reported their various stages of implementation at the time of the study (Table 1). Shi’s^9^ convenience sampling process was utilised to randomly select participants from each of the laboratories. Participants included laboratory managers and staff; participation was voluntary. Eleven participants from each lab (*N* = 44) completed the demographic survey and interview questions.

**TABLE 1:** Vietnamese hospitals participating in this study (2017).

Hospital identification	Hospital level	Completed QMS training programme	Laboratory status at time of study (2017)
H1	City	Yes	ISO 15189 accredited for laboratory tests, participating in EQA and QC programmes
H2	City	Yes	In the process of ISO accreditation for laboratory tests, participating in QC and EQA programmes
H3	City	Yes	QMS has not been successfully implemented, challenges include infrastructure, administration support, staff turnover
H4	District	Yes	Successfully implemented QMS in 2016. Working towards ISO 15189 accreditation

*Source*: Robinson CD. A multi case analysis of critical success factor in Vietnam laboratories implementing quality management systems to earn international accreditation (dissertation). 2018; Mount Pleasant, MI: Central Michigan University

EQA, external quality control; QC, quality control; QMS, quality management system; ISO, International Standardization Organization.

### Study design

The study was descriptive and employed a mixed design utilising qualitative and quantitative methods. Qualitative data were collected from staff employed in the study’s laboratories currently implementing a QMS project. Qualitative data collected from responses to the semi-structured interview provided participant insight to answer ‘why’ their laboratory scores did or did not improve over time. During the interview, participants were asked to list the top five factors they felt were most important in meeting QMS project goals and improving scores. For added clarity, each was asked to define the factors they listed.

A quantitative demographic survey was used to gather participant data such as age, gender and education levels.

With no previous CSF studies found related to QMS projects and medical laboratories, QMS experts from three countries outside of Vietnam served as benchmark panellists.

### Benchmark panel of laboratory quality management system experts

To validate this study’s findings, a panel of three experts from Kenya, Tanzania and Ukraine agreed to serve as benchmark experts and review the Vietnam study findings. Each was a practising medical laboratory scientist experienced in the subject matter, that is, laboratory quality in resource-limited countries, QMS, training and Stepwise Laboratory Quality Improvement Process Towards Accreditation checklist. The expert panellists endorsed the QMS implementation methods used in the Vietnamese study as those similarly used in their respective countries.

### Statistical analysis

The researchers used content analysis^10^ to review and sort all responses (220 listed factors) into exhaustive and mutually exclusive categories. Ten categories were identified. After identifying the content categories, the researcher and assistants sorted each of the factors into one of the content categories. Cohen’s Kappa statistic was used to measure inter-coder reliability between the researchers. This statistic provides a quantitative measure of reliability for two or more raters coding or sorting the same thing, corrected for how often the raters may agree by chance.

Applying frequency percentages, the top five categories were identified (Table 2). Though not the aim of the study, barriers were also identified using the same frequency percentage calculations. To look for bias between participants’ interview responses and demographic survey results, the chi-square test was applied. The same statistical analyses were applied to the data collected from the three expert panellists.

**TABLE 2:** Top five critical success factor categories identified during the data content analysis.

Rank	Critical success factor
1	Staff knowledge of quality management system
2	Lab manager leadership knowledge and skills
3	Staff motivation to change process
4	Mentorship
5	Hospital administration support

*Source*: Robinson CD. A multi case analysis of critical success factor in Vietnam laboratories implementing quality management systems to earn international accreditation (dissertation). 2018; Mount Pleasant, MI: Central Michigan University

## Results and discussion

Applying Cohen’s Kappa statistic, reliability values were greater than 0.85, indicating excellent reliability between the researchers in coding and sorting the CSFs.^11^ Likewise, the chi-square test found no relevant bias between the demographic responses and the factors listed by the participants.

Five top CSFs were identified from the Vietnamese participants’ responses via content analysis by the researchers.^12^ The strength of this study lies in the close alignment of the identified top five CSFs by the researchers and the expert panel after the analysis of participants’ responses. The top five success factors identified from the Vietnamese study and the expert panellists were identical although the individual rankings varied between the top five positions (Table 3).

**TABLE 3:** Comparative ranking of critical success factors between the Vietnam study and expert panel.

Vietnam study	QMS expert 1	QMS expert 2	QMS expert 3
1. Staff knowledge of QMS	1. Staff knowledge of QMS	1. Staff knowledge of QMS	1. Staff knowledge of QMS
2. Lab manager leadership	2. Staff motivation to change process	2. Staff motivation to change process	2. Hospital administration support
3. Staff motivation to change process	3. Hospital administration support	3. Mentorship	3. Staff motivation to change process
4. Mentorship	4. Lab manager leadership	4. Lab manager leadership	4. Lab manager leadership
5. Hospital administration support	5. Embedded mentorship	5. Hospital administration support	5. Mentorship

*Source*: Robinson CD. A multi case analysis of critical success factor in Vietnam laboratories implementing quality management systems to earn international accreditation (dissertation). 2018; Mount Pleasant, MI: Central Michigan University

QMS, Quality management system.

The number one CSF identified was staff knowledge of QMS. Specifically, all groups felt strongly that continuing education was crucial to ensuring current and new staff received QMS training and skills. Staff participants reported they wanted to improve the quality of their laboratories, but felt they lacked QMS knowledge to engage in the improvement processes. QMS knowledge included specific steps to follow when implementing a new task, why the specific task was important to test result accuracy and the importance of quality control monitoring. Although QMS was defined in the training, staff felt frequent reminders explaining the QMS concept, how and why QMS would improve their laboratory’s quality, and the benefit to patients would be beneficial and motivational. Without continuing education, participants reported staff turnover often left the laboratory without knowledgeable staff to continue the implementation process. Laboratory manager leadership was ranked as CSF number two by the study participants, whereas the expert panel ranked laboratory manager leadership as CSF number four. Staff commitment to the change process was the third CSF by the study participants; however, with the experts its ranking varied with expert #3 ranking it as third, and expert #1 and expert #2 ranking it as second.

Mentorship, as a CSF, was in varying positions between all groups. The variation in ranking may be due to many types of mentorship options. Expert #1 specifically listed embedded mentorship, whereas the others simply listed ‘mentorship’. Often, due to time constraints with both the laboratory staff and mentor, email communication emerged as a valuable part of the mentorship package. Discussing the mentorship experience at their laboratories, study participants agreed on the value of the mentorship without regard to whether the mentor visited the laboratory weekly or monthly. Several participants commented that their mentors provided motivation and quick responses to their questions on QMS implementation, often keeping them moving forward in implementation.

Without a mentor, resource staff indicated improvement projects stalled and were often discontinued. Previous articles suggest that embedded and longer mentorships result in better outcomes for the laboratories, although this was not the finding in this study.^5,6,7^

Hospital administration support received a wide range of scores. Hospital support was defined as either financial support for QMS resources or hospital-wide recognition for staff efforts. Even without financial support, staff reported recognition from administrators and other hospital staff to be motivational. Based on comments from the study participants, all agreed hospital support was critical but often lacking. This may account for the fifth-place ranking of hospital administration support.

The panel of experts endorsed the implementation methods employed by the Vietnamese laboratories. Methods and processes included QMS training, baseline assessments, development of a strategic plan and improvement projects to meet the strategic plan objectives (gaps).

Previous data reported quantitative outcome information based on individual laboratory assessments (Stepwise Laboratory Quality Improvement Process Towards Accreditation scores in each of 12 sections and a total score). Current and active involvement throughout the QMS implementation process made these participants uniquely qualified to share their experiences as well as identify those factors considered critical to QMS implementation success. Combining qualitative data from this study with previous quantitative findings offers valuable information to laboratory managers explaining ‘why’ implementation or improvements often stalled and failed to move forward.

One of the factors mentioned by both participants and experts is a perceived, or real, lack of knowledge by both laboratory managers and laboratory staff on QMS. The expert panellists confirmed a similar knowledge deficit of QMS principles and skills in staff attempting to implement unfamiliar processes in their laboratories.

Managers reported that they had received some management training but felt they would benefit from additional training related to staff orientation, conflict management, and quality control. Study participants at all laboratories said the formation of teams greatly improved both morale and motivation.

One interview question asked participants to list gaps identified during their project implementation as well as training they felt would strengthen their ability to successfully implement QMS and continue moving towards ISO 15189 accreditation (Table 4). With significant changes in daily laboratory functions due to QMS implementation, repetitive short training sessions for new and current staff seem reasonable to provide support and motivation until the changes become a normal daily routine.

**TABLE 4:** Examples of staff reported knowledge gaps and requests for training (2017).

Lack of knowledge	Requests for training
What a quality management system is.	Design an evaluation form to measure staff competency
How to determine the minimum and maximum reagent stock levels	Interpreting proficiency test results and determining root cause analysis
How to use the Plan, Do, Check, Act cycle to plan improvement projects	Quality indicators and how to set and monitor monthly goals
How to write different types of standard operating procedures	Equipment or method validation procedures
How to write a safety and quality manuals from scratch	Creating an orientation checklist
Quality assurance and quality control procedures and interpretation	Resolution of staff conflict
Organise and conduct staff meetings and purpose of minutes	Quality control procedures and interpretation of errors

*Source*: Robinson CD. A multi case analysis of critical success factor in Vietnam laboratories implementing quality management systems to earn international accreditation. 2018; Mount Pleasant, MI: Central Michigan University

C.D., Interview questions (2017).

### Limitations

This study focused on a small number of laboratories located in Vietnam. A larger study would offer more insight into CSFs that affect medical laboratory QMS success.

Asking study participants to make a forced ranking by only listing one CSF for each position is another limitation. Participants were unable to give equal rank to two CSFs.

### Conclusion

This study is the first to identify CSFs for the global medical laboratory sector. Equipped with QMS training, and now CSFs, managers can more precisely focus their time, resources and implementation strategy to address staff needs and move their implementation plan forward.

Utilising CSFs, implementation success should exhibit less variability between laboratories, and QMS projects should begin to move forward. Data from this study may strengthen QMS success throughout Vietnam and serve as a guide for managers in over 1000 laboratories in low-income and middle-income countries implementing QMS.^4,13^ Laboratory managers and staff considering implementation of a QMS as a pathway to improving quality aspects of their laboratory would benefit from the results of this study.
